# Opsin‐Free Activation of Bmp Receptors by a Femtosecond Laser

**DOI:** 10.1002/advs.202308072

**Published:** 2024-01-18

**Authors:** Manjun Xu, Haipeng Wang, Xiaoying Tian, Bingyi Li, Shaoyang Wang, Xiaohui Zhao, Hao He

**Affiliations:** ^1^ School of Biomedical Engineering Shanghai Jiao Tong University Shanghai 200031 China; ^2^ School of Biomedical Engineering Hainan University Haikou 570228 China

**Keywords:** BMP receptors, conformational change, opsin‐free activation, signaling pathways, two‐photon excitation

## Abstract

Bone morphogenetic protein (BMP) signaling plays a vital role in differentiation, organogenesis, and various cell processes. As a member of TGF‐β superfamily, the BMP initiation usually accompanies crosstalk with other signaling pathways and simultaneously activates some of them. It is quite challenging to solely initiate an individual pathway. In this study, an opsin‐free optical method to specifically activate BMP receptors (BMPR) and subsequent pSmad1/5/8 cascades by a single‐time scan of a tightly‐focused femtosecond laser in the near infrared range is reported. Via transient two‐photon excitation to intrinsic local flavins near the cell membrane, the photoactivation drives conformational changes of preformed BMPR complexes to enable their bonding and phosphorylation of the GS domain in BMPR‐I by BMPR‐II. The pSmad1/5/8 signaling is initiated by this method, while p38 and pSmad2 are rarely perturbed. Based on a microscopic system, primary adipose‐derived stem cells in an area of 420  × 420 µm^2^ are photoactivated by a single‐time laser scanning for 1.5 s and exhibit pSmad1/5/8 upregulation and osteoblastic differentiation after 21 days. Hence, an opsin‐free, specific, and noninvasive optical method to initiate BMP signaling, easily accomplished by a two‐photon microscope system is reported.

## Graphical abstract

An opsin‐free, specific, and noninvasive optical method to initiate BMP signaling by a transient scanning of a tightly‐focused femtosecond laser in the near‐infrared range is reported. Via the two‐photon excitation to intrinsic local flavins near the cell membrane, the photoactivation drives conformational changes of preformed BMPR complexes to initiate the phosphorylation of the GS domain in BMPR‐I and pSmad1/5/8 cascades.

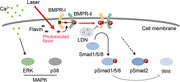

## 1 Introduction

Signaling pathways bridge extracellular stimuli and intracellular molecular cascades to realize cellular physiological functions and processes. Numerous signaling pathways compose a large network architecture with complex branches, feedback loops, and overlapping intersections. Bone morphogenetic proteins (BMPs) are a group of signaling molecules belonging to the transforming growth factor‐β (TGF‐β) superfamily, and play an essential role in a myriad of biological activities.^[^ ^1^ ^]^ Many cellular processes depend on BMP signaling for proliferation, differentiation, and migration. The differentiation of various types of progenitor cells is believed to be regulated by BMPs.^[^ ^2^, ^3^ ^]^ In some circumstances, BMP signaling also contributes to cell transitions. During embryogenesis, the BMP pathway drives ectoderm cell differentiation, establishes the dorsal‐ventral axis, and is prominently involved in mesoderm formation and cardiac development.^[^ ^4^, ^5^ ^]^ Organogenesis, including bone formation,^[^ ^6^ ^]^ gastrointestinal development,^[^ ^7^ ^]^ and angiogenesis,^[^ ^8^ ^]^ is the most well‐known function of BMP signaling.

BMP ligands bind to two types of transmembrane receptors, BMPR‐I and BMPR‐II, which then activate the expression of specific genes.^[^ ^9^, ^10^ ^]^ In this process, crosstalk between downstream pathways is inevitable. Wingless and Int‐1 (Wnt), Hedgehog, and Notch pathways usually coordinate tissue homeostasis together with BMP.^[^ ^11^, ^12^, ^13^ ^]^ There exist spontaneous preformed complexes (PFCs, composed of both BMPR‐I and BMPR‐II) and BMP ligand‐induced signaling complexes (BISCs) of BMPRs in the cell membrane.^[^ ^14^ ^]^ The typical BMP‐Smad transduction is initiated by the binding of BMP ligands with BMPR PFCs. After that, BMPR‐II trans‐phosphorylates the segment with a sequence of SGSGSG (GS domain) in BMPR‐I. BMPR‐I then phosphorylates the R‐Smads (Smad1/5/8) that associate with the co‐Smad (Smad4) and activate the expression of target genes.^[^ ^15^, ^16^, ^17^ ^]^ The activated ligand‐receptor complexes can also stimulate the mitogen‐activated protein kinase (MAPK/p38) pathway.^[^ ^18^ ^]^ The other type of BMP signaling, BISC, is activated if BMP dimers directly bind to high‐affinity BMPR‐I dimers, which subsequently recruits BMPR‐II and finally initiates MAPK/p38 pathways.^[^ ^19^, ^20^ ^]^ It is quite challenging to activate an individual BMP pathway to precisely encode specific downstream responses.

In this study, we report an opsin‐free optical method to specifically activate the conformational change of BMPR PFCs, which consequently initiates the Smad cascades transduction, without BMPs or any other exogenous molecules. We propose that this technology has good potential for noninvasive and precise control of BMP signaling and the disentanglement of BMP pathways.

## 2 Results

### 2.1 Photoactivation of BMP Pathway

**Figure 1:**
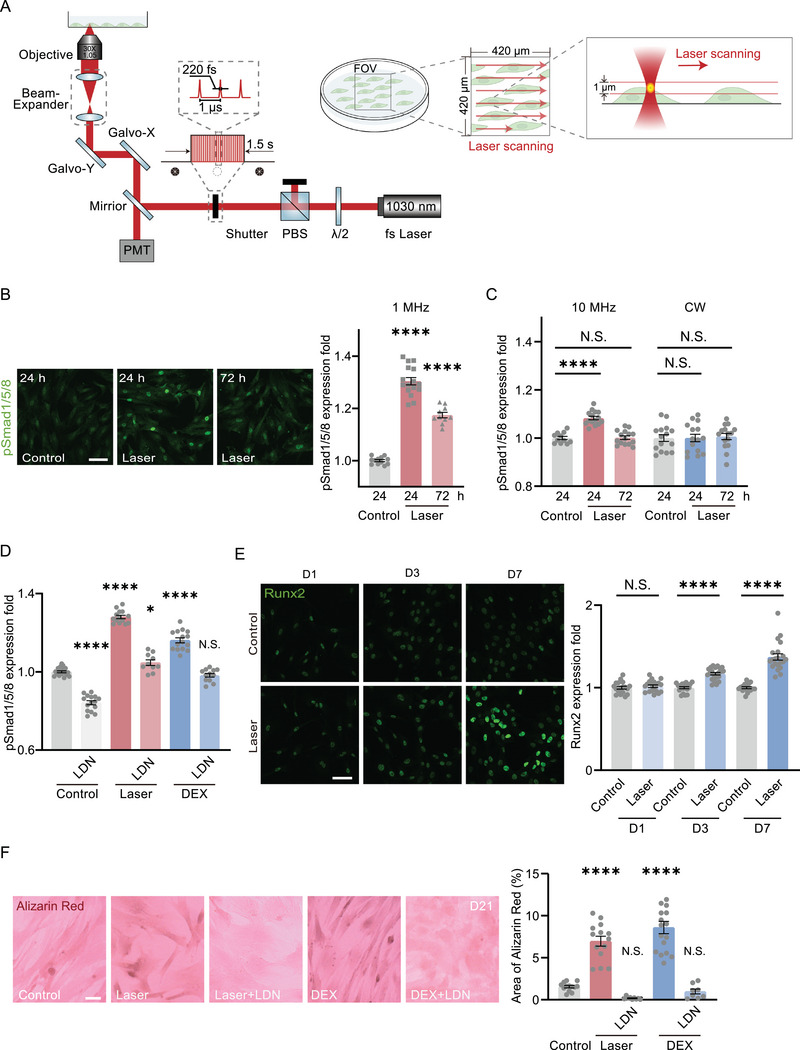
BMP photoactivation directly by a transient femtosecond‐laser stimulation. A) Optical design of the photoactivation to cells. The 1030‐nm femtosecond laser (220 fs, 1 MHz, 8 mW) was coupled to an inverted microscope and focused precisely on the cell membrane by an objective (30  ×, oil immersed, N.A. = 1.05). The photoactivation was controlled by a shutter and galvo‐mirrors for a single‐time 1.5‐s scanning of the laser in a predefined region in the X‐Y plane. λ/2, the half‐wave plate; PBS, polarization beam splitter; FOV, field of view. B) The immunofluorescence of phosphorylated Smad 1/5/8 (pSmad1/5/8) in ADSCs 24 h (n = 16 fields in 4 independent trials) and 72 h (n = 10 fields in 3 independent trials) after photostimulation (1 MHz unless specifically stated). Control: ADSCs cultured for 24 h without any treatment (n = 12 fields in 3 independent trials). Scale bar: 80 µm. Right panel: quantified pSmad1/5/8 immunofluorescence level from the Left. C) The quantified immunofluorescence of pSmad1/5/8 in ADSCs 24 and 72 h after 10 MHz femtosecond laser stimulation (Control, n = 11 fields in 3 independent trials; Laser, n = 15 fields in 3 independent trials for both) and continuous‐wave laser stimulation at the same power and wavelength (n = 15 fields in 3 independent trials for each group). D) The quantified immunofluorescence of pSmad1/5/8 in ADSCs 24 h after photostimulation (Laser, n = 14 fields in 3 independent trials; LDN, n = 10 fields in 3 independent trials) and DEX (10 nm) treatment (DEX, n = 15 fields in 4 independent trials; LDN, n = 12 fields in 3 independent trials), with or without the presence of BMPR‐I inhibitor LDN193189 (1 µm), respectively. Control: ADSCs without any treatment (n = 18 fields in 5 independent trials) or with LDN193189 (1 µm) treated (n = 15 fields in 3 independent trials). E) The immunofluorescence of Runx2 in ADSCs 1 day (Control, n = 18 fields in 3 independent trials; Laser, n = 19 fields in 3 independent trials), 3 days (Control, n = 19 fields in 3 independent trials; Laser, n = 23 fields in 3 independent trials) and 7 days (Control, n = 17 fields in 3 independent trials; Laser, n = 21 fields in 3 independent trials) after photoactivation. Scale bar: 50 µm. Right panel: quantified Runx2 immunofluorescence level from the Left. F) The Alizarin red S test in ADSCs cultured for 21 days from the groups of Control (n = 13 fields in 3 independent trials), Laser (photostimulation at Day 0, with (n = 9 fields in 3 independent trials) or without (n = 14 fields in 4 independent trials) LDN193189 (1 µm)), and DEX (10 nm, with (n = 8 fields) or without (n = 17 fields in 4 independent trials) LDN193189 (1 µm)). Scale bar: 20 µm. Comparison was taken with Control group without any treatment. ^*^ *P* < 0.05, ^****^ *P* < 0.0001, by two‐tailed unpaired t‐test and one‐way ANOVA analysis corrected by Dunnet's post‐hoc. N.S., no significant difference.

At first, we demonstrated a transient two‐photon excitation to BMPRs by a femtosecond laser in the near infrared (NIR) range was sufficient to activate the BMP‐Smad signaling. The two‐photon excitation was accomplished by scanning of the laser on the cell membrane based on a microscopic system (**Figure** **1**A). A femtosecond laser at 1030 nm (220 fs, 1 MHz, 8 mW) was coupled to an inverted confocal microscope and focused by an objective (30  ×, oil immersed, N.A. = 1.05). The laser focus was around 1 µm in diameter and controlled by galvo‐mirrors and a mechanical shutter. The cells in the field of view (FOV, 420  × 420 µm^2^) were photoactivated by a single‐time X‐Y plane scanning for 1.5 s, controlled by the galvo‐mirrors. The shutter was only open during the photoactivation, synchronized with the laser scanning frame. In this scheme, the laser focus was vertically tuned to be located at the upper part of those adherent cells. During the scanning of the laser in the FOV, the laser focus swept past the cell membrane on top and activated BMPRs there.

The femtosecond laser output was set at 1030 nm because photons with such low single‐photon energy could hardly generate any linear photochemical reactions.^[^ ^21^, ^22^, ^23^ ^]^ The ultra‐low repetition rate (1 MHz) minimized the thermal deposition between femtosecond pulses (the duty cycle was only 2  × 10^−7^), ensuring that the thermal effect of the laser minimally heated cells. In our experiments, primary adipose‐derived stem cells (ADSCs) were extracted from rats and cultured in petri dishes to demonstrate BMP‐Smad activation using this method. The ADSCs adherent on the glass slide of the dishes suffered a single‐time laser scanning and were then continuously cultured for several days. We observed a significant upregulation of phosphorylated Smad 1/5/8 (pSmad1/5/8) in the photostimulated ADSCs after 24 and 72 h (Figure 1B). To guarantee the thermal effect of the laser did not contribute to these results, the repetition rate of the femtosecond laser was then tuned to 10 MHz at the same mean power to provide higher thermal deposition but much lower peak power of pulses.^[^ ^24^ ^]^ The pSmad1/5/8 level increased slightly 24 h after photoactivation but returned to normal after 72 h probably due to the low peak power and thus low two‐photon excitation efficiency by the laser with 10 MHz repetition rate (Figure 1C). A continuous‐wave laser at 1030 nm with the same power, capable of continuously heating cells to provide the highest thermal deposition, was also used to activate the ADSCs for comparison. However, no upregulation of pSmad1/5/8 was observed (Figure 1C). Hence, the thermal effect of the laser did not contribute to BMP‐Smad activation.

The presence of the specific inhibitor of BMPR‐I, LDN193189, could significantly suppress the pSmad1/5/8 level activated by laser (Figure 1D), suggesting its upregulation was through BMP signaling pathway. To further demonstrate that the photoactivation of BMP pathway was functional, the photoactivated ADSCs were further cultured for 21 days. The osteoblastic marker, Runx2, was significantly higher in those ADSCs 7 days after photoactivation (Figure 1E). We confirmed that those ADSCs finally differentiated into osteoblasts on Day 21 (Figure 1F). The presence of LDN193189 in the transient photoactivation process could inhibit osteoblastic differentiation. These results were consistent with the positive control by the osteoblastic inducer, Dexamethasone (DEX) (Figure 1D,F). Therefore, the transient single‐time femtosecond laser scanning could activate BMP signaling pathway without any exogenous molecules or optogenetic engineering.

**Figure 2:**
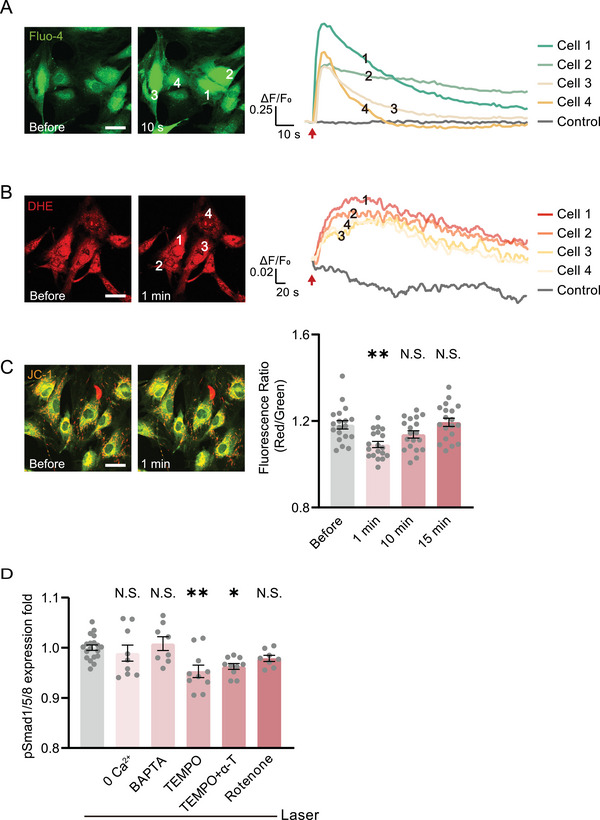
The photoactivated BMP signaling is not initiated by cellular acute responses to photoactivation stress. A,B) Fluorescence and quantified kinetic plots of cellular Ca^2+^ and ROS in ADSCs indicated by Fluo‐4/AM and DHE after photoactivation, respectively. Arrows: photostimulation location. C) Fluorescence and quantified MMP level before and after photoactivation in ADSCs indicated by JC‐1 (n = 19 cells). Comparison was taken with the group before photostimulation. D) The quantified pSmad1/5/8 immunofluorescence 24 h after photostimulation, incubated with Ca^2+^‐free buffer (n = 9 fields in 3 independent trials), intracellular Ca^2+^ chelator BAPTA‐AM (10 µm, n = 8 fields), ROS scavengers TEMPO (200 µm, n = 10 fields in 3 independent trials), both TEMPO and α‐tocopherol (100 µm, n = 10 fields in 3 independent trials), and mitochondria electron transport chain inhibitor rotenone (10 µm, n = 8 fields), compared with the group suffered with photostimulation without any drugs (n = 19 fields in 3 independent trials). ^*^ *P* < 0.05, ^**^ *P* < 0.01, by one‐way ANOVA analysis corrected by Dunnet's post‐hoc. N.S., no significant difference. Scale bar: 40 µm.

We then examined if the acute responses of ADSCs to the photoactivation stress induced the BMP signaling.^[^ ^25^, ^26^, ^27^, ^28^, ^29^ ^]^ The intracellular Ca^2+^ showed an immediate rise after photoactivation but recovered to normal after a few seconds (**Figure** **2**A). The reactive oxygen species (ROS) also exhibited a moderate increase and soon declined (Figure 2B). The mitochondrial membrane potential was partially depolarized and recovered after 15 min (Figure 2C). These results suggest that the photoactivation did not impose significant stress on the cells. We then examined if these responses contributed to the initiation of BMP signaling. There was no suppression of the pSmad1/5/8 level in the photostimulated ADSCs in the Ca^2+^‐free buffer or with intracellular Ca^2+^ chelated (Figure 2D). The inhibition of the electron transport chain in mitochondria did not suppress the photoactivated upregulation of pSmad 1/5/8 either. The scavenging of ROS decreased the pSmad level to 95% of the photoactivated level (Figure 2D). These results suggested that the direct stress responses to photostimulation were not the dominant factor in BMP signaling initiation.

### 2.2 Photoactivated Conformational Change of BMP Receptors Initiates Smad1/5/8 Cascades

**Figure 3:**
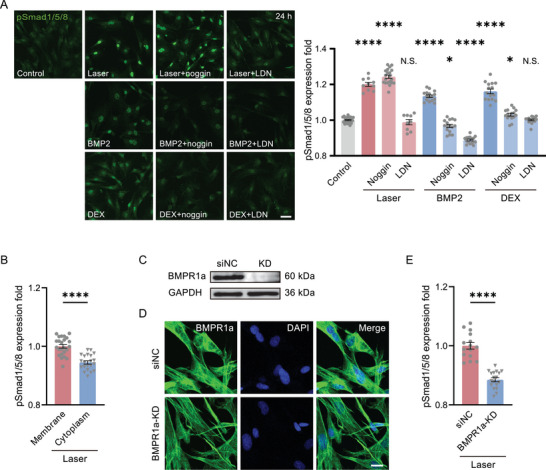
The femtosecond‐laser photostimulation activates BMP signaling specifically through BMPRs. A) The immunofluorescence of pSmad1/5/8 24 h after photostimulation (Laser, n = 10 fields in 3 independent trials; noggin, n = 20 fields in 4 independent trials; LDN, n = 9 fields in 3 independent trials), BMP2 (100 ng mL^−1^) treated (n = 16 fields in 3 independent trials for each group) and DEX (10 nm) treated (DEX, n = 15 fields in 3 independent trials; noggin, n = 16 fields in 3 independent trials; LDN, n = 15 fields in 3 independent trials), respectively, with or without BMPR‐I inhibitor LDN193189 (1 µm) and BMP Ligand antagonist noggin (100 ng mL^−1^), compared with Control group (n = 24 fields in 6 independent trials). Scale bar: 50 µm. Right panel: the quantified pSmad level from the fluorescence in the Left. B) The quantified pSmad1/5/8 level 24 h after photostimulation located in the cell membrane (n = 24 fields in 3 independent trials) and cytoplasm (n = 21 fields in 3 independent trials). C) Western blot of BMPR1a in the negative control (siNC, transfected with an invalid siRNA) and BMPR1a‐KD ADSCs. D) Immunofluorescence images of BMPR1a in siNC and BMPR1a‐KD ADSCs. Scale bar: 20 µm. E) The quantified pSmad1/5/8 level in siNC (n = 15 fields in 3 independent trials) and BMPR1a‐KD ADSCs (n = 18 fields in 3 independent trials) 24 h after photostimulation, normalized by the level in siNC group. ^*^ *P* < 0.05, ^****^ *P* < 0.0001, by two‐tailed unpaired *t*‐test and one‐way ANOVA analysis corrected by Dunnet's post‐hoc. N.S., no significant difference.

We then clarified how the BMP signaling was directly initiated by laser in the absence of BMP ligands. Two different inhibitors of BMPRs, Noggin and LDN193189, which respectively inhibit the extracellular binding site of BMPRs for ligands and the intracellular phosphorylation site of BMPRs, were used to treat cells respectively. These two inhibitors suppressed pSmad1/5/8 in the positive control by ligand BMP2, or DEX. However, only LDN193189 inhibited BMP signaling in the photoactivated ADSCs (**Figure** **3**A). This result implied the photoactivation directly induced the intracellular phosphorylation of BMPRs instead of activating the extracellular BMP ligand binding sites. As a comparison, when the laser focus was moved down into the cytoplasm, the pSmad1/5/8 level was significantly lower than when activated by laser scanning on the cell membrane (Figure 3B). To further confirm that the photoactivation target was BMPRs, the BMPR1a was knocked down (KD), verified by Western Blot and immunofluorescence microscopy (Figure 3C,D). Then, in those KD ADSCs, the pSmad1/5/8 upregulation after photoactivation was greatly reduced. (Figure 3E). In this regard, we speculated the transient femtosecond laser scanning directly activated the intracellular phosphorylation site of BMPRs.

**Figure 4:**
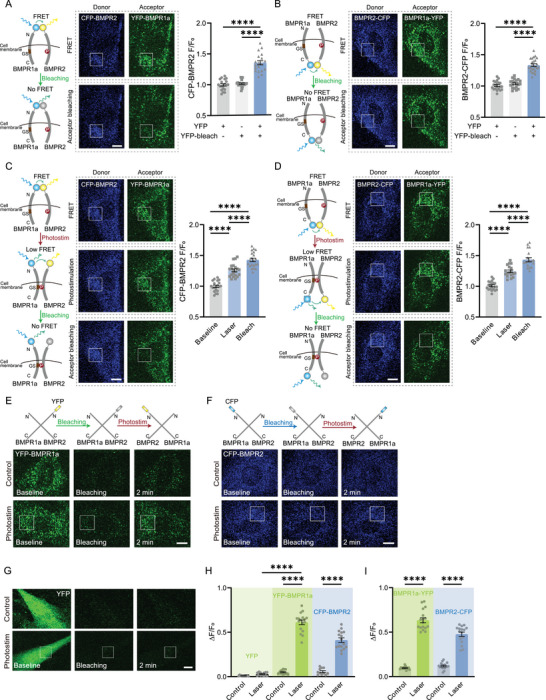
The conformational change of BMPRs initiated by photoactivation. A) CFP‐BMPR2 and YFP‐BMPR1a FRET test indicated by CFP fluorescence by YFP photobleaching. Controls: ADSCs without YFP‐BMPR1a transfected, or without YFP photobleaching (n = 19‐20 cells of each group in 3 independent trials). Boxes, acceptor photobleaching region. Scale bar: 10 µm. Right panel: quantified CFP fluorescence after the intense 473‐nm laser scanning for YFP photobleaching. B) FRET test by BMPR2‐CFP and BMPR1a‐YFP before and after YFP photobleaching (n = 20‐24 cells of each group in 3 independent trials). Boxes: photobleaching region. Scale bar: 10 µm. Right panel: quantified CFP fluorescence after photobleaching. C) FRET test by CFP‐BMPR2 and YFP‐BMPR1a after photostimulation and subsequent photobleaching of YFP (acceptor) (n = 21 cells in 3 independent trials). Boxes, photostimulation, and photobleaching region. Scale bar: 10 µm. Right panel: quantified CFP fluorescence after photostimulation and then photobleaching. D) Similar FRET test by BMPR2‐CFP and BMPR1a‐YFP (n = 18 cells in 3 independent trials). Scale bar: 10 µm. (E,F) Pre‐bleached YFP‐BMPR1a E) and CFP‐BMPR2 F) recovered after photostimulation. Boxes, photostimulation region. Scale bar: 10 µm. G) Pre‐bleached YFP expressed in whole cells could not recover after photostimulation. Boxes, photostimulation region. Scale bar: 10 µm. H) The quantified recovered ratio of YFP fluorescence (Control, n = 12 cells in 3 independent trials; Laser, n = 13 cells in 3 independent trials), YFP‐BMPR1a (Control, n = 15 cells in 3 independent trials; Laser, n = 17 cells in 4 independent trials) and CFP‐BMPR2 (Control, n = 11 cells in 3 independent trials; Laser, n = 16 cells in 3 independent trials). I) The quantified recovered ratio of BMPR1a‐YFP fluorescence (Control, n = 14 cells in 3 independent trials; Laser, n = 17 cells in 3 independent trials) and BMPR2‐CFP (Control, n = 17 cells in 3 independent trials; Laser, n = 16 cells in 3 independent trials). ^****^ *P* < 0.0001 by two‐tailed unpaired *t*‐test.

We verified this hypothesis by labeling the N‐terminus of BMPR2 and BMPR1a with Cyan fluorescence protein (CFP) and Yellow fluorescence protein (YFP) respectively to examine the conformational change of these two receptors by measuring the Foster Resonance Energy Transfer (FRET) signals. Initially, the CFP‐BMPR2 exhibited significantly enhanced fluorescence when the YFP‐BMPR1a was bleached (by continuous 473 nm laser scanning) (**Figure** **4**A), suggesting that the BMPR PFCs had already bound together at the N terminus. This binding caused the CFP fluorescence to rise after YFP bleaching, which stopped the CFP‐YFP FRET, and the transferred energy was thus returned to CFP. The 473‐nm laser, instead of green lasers at the YFP‐excitation, was used here to provide pure oxidative stress and avoid the reversible bleaching of YFP or photoactivation of CFP that could cause false‐positive signals in FRET microscopy.^[^ ^30^, ^31^ ^]^ The CFP‐BMPR2 fluorescence (without YFP‐BMPR1a transfected) treated with such 473‐nm laser scanning did not show any difference (Figure 4A). We also checked the C‐terminus of BMPRs using this method and observed the same results (Figure 4B). These data suggest that BMPR PFCs had bound together at both N─ and C─ terminus, but the GS domain of BMPR1a was not phosphorylated by BMPR‐II before photoactivation.

We then employed this method to assess the spatial distance between BMPRs during the photoactivation process. After photoactivation, the CFP‐BMPR1a showed increased fluorescence, indicating a decreased FRET and thus the separation of the N‐terminus of BMPR1a and BMPR2 (Figure 4C). An additional increase of CFP fluorescence could be observed when YFP was subsequently bleached (Figure 4C). Hence the distance between the N‐terminus of BMPR1a and BMPR2 was still < 30 nm, allowing for partial FRET. Such N‐terminus detachment of BMPRs was consistent with that induced by DEX (Figure S1, Supportingg Information). Similarly, the C‐terminus of BMPRs exhibited lower FRET signals after photoactivation and their distance was still quite close for partial FRET (Figure 4D). Given the upregulation of pSmad1/5/8 after photoactivation confirmed the phosphorylation of the GS domain in BMPR1a by BMPR2, in this regard, the photoactivation induced the detachment of both C‐ and N‐ terminuses of BMPRs but the GS domain of BMPR‐I binding with BMPR‐II, as illustrated in Figure 4C,D.

To rule out the possibility of photoconversion or photoswitching^[^ ^32^, ^33^ ^]^ of fluorescent proteins (FPs) after photobleaching, ADSCs were transfected solely with YFP‐BMPR1a (no CFP transfected). The YFP‐BMPR1a was pre‐bleached by the 473‐nm laser scanning in the entire cell. Then, a subcellular region was stimulated with a single‐time femtosecond laser scanning. The YFP fluorescence immediately recovered partially (Figure 4E,H). The pre‐bleached CFP‐BMPR2 exhibited similar fluorescence recovery after the femtosecond‐laser photostimulation (Figure 4F,H). However, if the cells were transfected with only YFP (not fused with BMPRs) and the YFP was bleached, its fluorescence could not recover after photostimulation (Figure 4G,H). The fluorescence recovery of FPs fused with BMPRs induced by femtosecond‐laser photostimulation suggested the reorientation and transition of BMPRs.^[^ ^34^ ^]^ The bleaching could not turn down all FPs due to their anisotropy.^[^ ^35^, ^36^ ^]^ After photostimulation, the reorientation and transition of BMPRs exposed the remaining FPs to fluorescence excitation. Consistently, if the CFP and YFP were fused with the C‐terminus of BMPR2 and BMPR1a, respectively, and transfected into cells separately, the pre‐bleached FPs partially recovered after the single‐time femtosecond‐laser photostimulation (Figure 4I and Figure S2, Supportingg Information).

**Figure 5:**
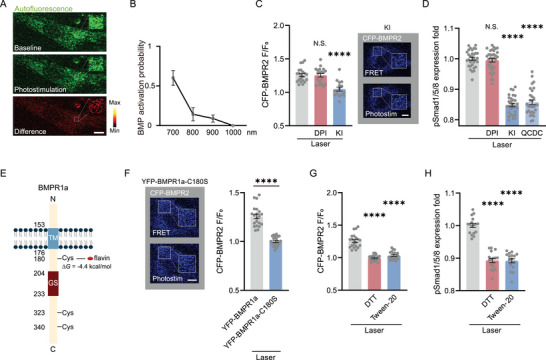
Photoreceptors in the photoactivation of BMPRs. A) Autofluorescence of flavin molecules before and after photostimulation, with their difference shown in the heat map (n = 5 cells). Boxes: the photostimulation region. B) Spectrum of BMP photoactivation probability (n = 22–25 cells at each wavelength in 3 independent trials). C) FRET test indicated by CFP fluorescence in groups of photostimulation without any other treatments (n = 21 cells in 3 independent trials), in the presence of DPI (10 µm, n = 19 cells in 3 independent trials) and KI (25 mm, n = 16 cells in 3 independent trials). Insert: FRET test with KI. Boxes, photostimulation region. D) The quantified pSmad1/5/8 fluorescence 24 h after photostimulation in the presence of DPI (10 µm, n = 34 fields in 4 independent trials), KI (25 mm, n = 30 fields in 4 independent trials), QCDC (10 µm, n = 30 fields in 4 independent trials) and without any drugs (n = 33 fields in 4 independent trials). E) Simulated binding energy of photoexcited flavin with each animo site in BMPR1a (rats). The most probable binding site, C180, presented a potential binding energy *ΔG* = −4.4 kcal mol^−1^ with photoexcited flavin. TM: transmembrane region. F) FRET test by CFP‐BMPR2 and YFP‐BMPR1a‐C180S after photostimulation (n = 21 cells in 3 independent trials for both mutation and control group). Left: FRET signals of YFP‐BMPR1a‐C180S. Boxes, photostimulation region. G) FRET test indicated by CFP after photostimulation in the presence of DTT (2 mm) and Tween‐20 (0.025%, v/v, 20 cells in 3 independent trials for both), compared with the group without any drugs (n = 21 cells in 3 independent trials). H) The pSmad1/5/8 level 24 h after photostimulation in the presence of DTT (2 mm) and Tween‐20 (0.025%, v/v), compared with the photostimulation group (n = 17 fields in 3 independent trials for each group). ^****^ *P* < 0.0001 by two‐tailed unpaired *t*‐test and one‐way ANOVA analysis corrected by Dunnet's post‐hoc. N.S., no significant difference. Scale bar: 10 µm.

We suspected flavin, the endogenous photosensitive molecules of cells, might be responsible for mediating the photoactivated conformational change of BMPRs.^[^ ^37^ ^]^ The autofluorescence of flavin^[^ ^38^ ^]^ in the photostimulation region, which directly indicated the local level of flavin molecules, was found significantly decreased after photoexcitation (**Figure** **5**A). More importantly, the BMP photoactivation efficiency spectrum was quite close to the two‐photon excitation spectrum of flavins^[^ ^39^ ^]^ (Figure 5B). These clues were further verified by using the specific inhibitors of free and bonded flavin separately. The inhibitor of free flavins, potassium iodide (KI), showed significant inhibition to the BMPRs activation, whereas the inhibitor of flavoproteins (bonded flavin) DPI did not present any inhibition to conformational changes of BMPRs (Figure 5C). This result was confirmed by testing the pSmad 1/5/8 level in the presence of KI, diphenyliodonium chloride (DPI), and quinacrine dihydrochloride (QCDC) (another specific inhibitor of free flavins) separately. We found KI and QCDC significantly suppressed the pSmad1/5/8 expression after photoactivation (Figure 5D). Therefore, cellular endogenous flavins were the key mediators of BMPRs photoactivation.

Previous studies suggested the photoexcited flavin could combine with cysteine (Cys) residues in proteins by forming a thioether bond which further generated hydrophobic cavities in the protein and mediated the formation of hydrophobic bonds between them.^[^ ^40^, ^41^ ^]^ Hence we verified whether the phosphorylation of the GS domain in BMPR‐I by the kinase domain in BMPR‐II followed this mechanism. We calculated the binding energy of each Cys in BMPR1a and found C180 was the most probable binding site with the highest potential energy of −4.4 kCal mol^−1^ with photoexcited flavin, which exactly located at the closest position to the GS domain (204‐233) (Figure 5E). If C180 in BMPR1a was mutant to serine (C180S), the photoactivation could not induce the conformational change of BMPRs (Figure 5F). We used Dithiothreitol (DTT) and low‐concentration Tween‐20 treatment to suppress the formation of thioether bonds (photoexcited flavins – Cys) and hydrophobic bonds (BMPR‐I – BMPR‐II), respectively. Each of both inhibited the BMPRs conformational changes activated by the femtosecond laser (Figure 5G). This result was finally confirmed with the suppressed pSmad 1/5/8 expression by them (Figure 5H). Hence the femtosecond laser excited cellular endogenous flavins located near the cell membrane which then mediated the phosphorylation of the GS site in BMPR‐I by BMPR‐II.

### 2.3 Specificity of BMP Photoactivation

**Figure 6:**
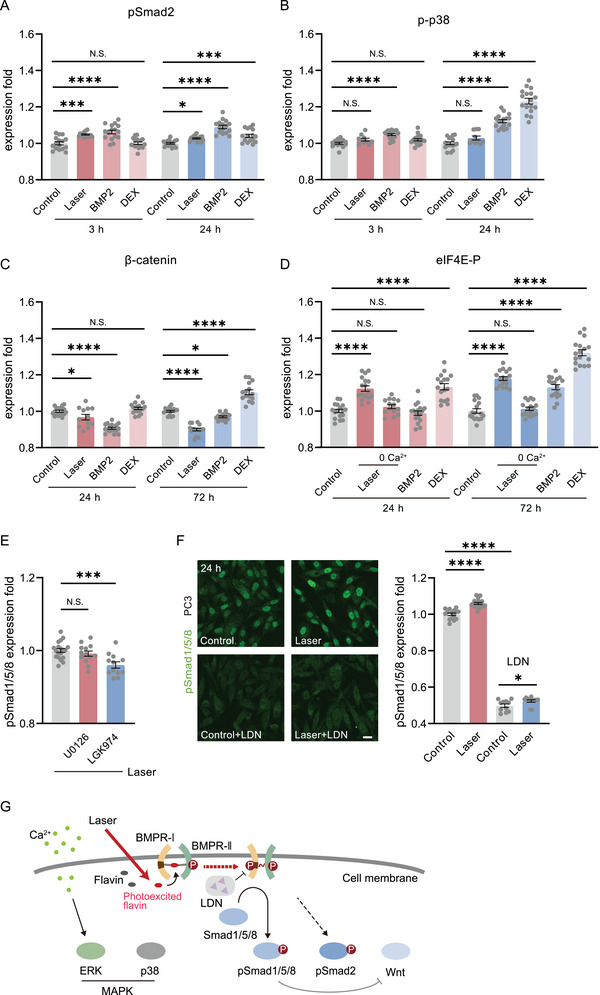
Specificity of BMP photoactivation. A) The phosphorylated Smad 2 (pSmad2) expression level in ADSCs 3 h and 24 h after photostimulation, BMP2 (100 ng mL^−1^) and DEX (10 nm) treated (n = 16 fields in 3 independent trials for each group). B) The phosphorylated p38 (p‐p38) level in ADSCs 6 h (Control, n = 15 fields in 3 independent trials; Laser, n = 10 fields in 3 independent trials; BMP2 and DEX, n = 18 fields in 3 independent trials for both) and 24 h (Control, n = 17 fields in 3 independent trials; Laser, n = 10 fields in 3 independent trials; BMP2 and DEX, n = 17 fields in 3 independent trials for both) after laser or drug‐treated. C) The β‐catenin level in ADSCs 24 h (Control, BMP2, and DEX, n = 17 fields in 3 independent trials for each; Laser, n = 12 fields in 3 independent trials) and 72 h (n = 15 fields in 3 independent trials for each group) after laser or drug‐treated. D) The phosphorylated eIF4E (eIF4E‐p) level in ADSCs 24 h and 72 h after photostimulation (with or without the depletion of intercellular Ca^2+^) and drug‐treated (n = 14 fields in 3 independent trials for 24 h after photostimulation with the depletion of intercellular Ca^2+^, n = 17 fields in 3 independent trials for others). E) The pSmad1/5/8 level in ADSCs 24 h after photostimulation, in the presence of U0126 (20 µm, n = 13 fields in 3 independent trials) and LGK974 (5 µm, n = 12 fields in 3 independent trials), compared with the group without any drugs (n = 19 fields in 4 independent trials). F) The immunofluorescence and quantified level of pSmad1/5/8 in PC3 cells 24 h after photostimulation, with (Control, n = 11 fields in 3 independent trials; Laser, n = 12 fields in 3 independent trials) or without (Control, n = 15 fields in 3 independent trials; Laser, n = 18 fields in 4 independent trials) the presence of LDN193189 (1 µm). Scale bar: 20 µm. G) Schematic diagram of the BMP photoactivation process. Comparison was taken with Control. ^*^ *P* < 0.05, ^***^ *P* < 0.001, ^****^ *P* < 0.0001, by two‐tailed unpaired *t*‐test. N.S., no significant difference.

We finally examined the photoactivation specificity of the BMP signaling pathway. At first, after photoactivation, we found pSmad‐2 was also upregulated but very slightly (5% at 3 h, 3% at 24 h, **Figure** **6**A). The p‐p38 did not show any upregulation in the laser‐treated group (Figure 6B). However, pSmad‐2 and p38 both upregulated significantly if the cells were treated with BMP2 or DEX (Figure 6A,B). The β‐catenin expression decreased significantly after photoactivation and BMP2 treated (Figure 6C), suggesting the inhibition of Wnt signaling, consistent with the inhibition effect to Wnt by the activation of osteoblast genes initiated by pSmad 1/5/8.^[^ ^42^, ^43^ ^]^ However, DEX induced upregulation of β‐catenin (Figure 6C). Interestingly, although the eIF4E‐P was upregulated by photoactivation, the activation of ERK was found due to the transient photoexcited Ca^2+^ rise in cells (Figure 6D).^[^ ^44^ ^]^ If intracellular Ca^2+^ was chelated, the eIF4E‐P overexpression was then suppressed. Therefore, the photoactivation solely activated the preformed binding of BMPR‐I and II (PFCs). The combination between BMPR‐I and II dimers (like BISCs that activated p38) could hardly be initiated by laser.

To further confirm these results, the inhibitors of ERK and Wnt, U0126 and LGK974, were used to treat cells during photoactivation, respectively. The ERK inhibition did not show any influence on photoactivated BMP, while Wnt inhibition slightly suppressed the pSmad 1/5/8 expression level (declined to 96%) (Figure 6E), as the crosstalk between them was notoriously complex.^[^ ^43^ ^]^ Hence, the photoactivation method activated BMP relatively specifically. Finally, we tested the photoactivation versatility in different cell lines. The photoactivation also upregulated pSmad 1/5/8 in PC3 cells while LDN193189 could greatly suppress it (Figure 6F). We summarized the BMP photoactivation process in Figure 6G.

## 3 Discussion

In this study, we report a photochemical technology by performing a single‐time transient NIR femtosecond‐laser scanning on the cell membrane to activate local BMPRs and initiate intracellular BMP signaling. The diffraction‐limited focusing and two‐photon absorption of endogenous flavin together produce the specificity of this technology. In our experiments, the spontaneous preformed BMPR complexes were activated by this photochemical excitation and predominantly initiated pSmad1/5/8 cascades. The combination of two pre‐separated BMPR dimers (BISCs) could hardly be driven by this method (Figure 6B,D). This result is consistent with the conformational change of BMPRs (Figure 4). The transient photoexcited flavin mediates the hydrophobic binding of BMPR PFCs and triggers the conformational change of BMPRs to expose the L45 loop of BMPR‐I which drives the phosphorylation of BMPR‐I and the fluorescence recovery of FPs fused on the BMPRs.^[^ ^45^, ^46^ ^]^ Therefore, this photochemical method activates BMPR PFCs and the pSmad1/5/8 cascades.

Although BMPRs are single‐pass transmembrane proteins, the conformational changes of them are quite complex after photoactivation. The BMPR PFCs presented in tetramer form, composed of a BMPR‐I dimer and BMPR‐II dimer. The C‐ and N‐ terminuses of BMPR‐I and BMPR‐II are originally close to each other, respectively, exhibiting a cross‐helical structure. After photoactivation, their C‐ and N‐ terminuses detach but the GS domain of BMPR‐I bonds with the kinase domain of BMPR‐II, probably by the twist of those two receptors. In this process, due to the anisotropy of FPs, some fused FP‐BMPRs that are originally perpendicular to the laser polarization remain un‐bleached, reorient along with BMPRs after photoactivation, and partially recover the fluorescence. If these recovered FPs are bleached again and photostimulated for the second time, their fluorescence can still partially recover (Figure S3, Supportingg Information).

In this study, the two‐photon excitation was used to avoid unspecific absorption and photodamage by blue lasers. The tightly focused NIR femtosecond laser confined the effective excitation solely inside the laser focus (located close to the cell membrane). The laser at 488 nm can theoretically work for this purpose. However, the unspecific absorption of cellular molecules to such high‐energy photons along the whole light propagation path induces dramatic photodamage and out‐of‐target excitation. In this regard, two‐photon excitation by NIR femtosecond laser holds both high excitation efficiency and specificity, providing a noninvasive and relatively specific activation of BMP in target cells.

## 4 Experimental Section

### Cell Culture

ADSCs were extracted from adipose tissues of the groin fat pads from SD rats at about postnatal day 7. The adipose tissue was washed in PBS, cut up, and then digested in 0.1% collagenase type I (Sigma–Aldrich, C0130) at 37°C for 30 min with shaking until the fat was emulsified. The digested solution was filtered through a 70 µm cell strainer (BD Bioscience) to obtain a single‐cell suspension and then centrifuged at 800 × g for 5 min. The supernatant containing adipocytes and debris was discarded. The cell pellet was immediately resuspended in BASIC MEM alpha culture medium (Gibco, C12571500BT) with 10% FBS (Gibco, 10099–141C) and 1% penicillin and streptomycin (Gibco, 15 140 122). The single cell suspension was transferred to a 100 mm culture dish (Corning) and incubated at 37°C with 5% CO_2_ for 72 h before the first change of the medium. Afterward, the medium was changed every two days. The cells were digested with TrypLE (Gibco, 12 605 010) and first passaged at the ratio of 1:2 or 1:3 when the cell density reached 80%−90%. All the ADSCs were used within the first 5 passages.

The osteogenesis differentiation medium was composed of 10 nm dexamethasone (DEX, D4902), 10 mm b‐glycerophosphate (G9422) and 50 mm L‐Ascorbicacid‐2‐phosphate (A8960, all from Sigma–Aldrich) in BASIC MEM alpha culture medium (ThermoFisher, C12571500BT) with 10% FBS (Gibco, 10270‐106) and 1% penicillin‐streptomycin (Gibco, 15 140 122). After incubation for 21 days, ADSCs were stained with Alizarin Red S (Sigma–Aldrich, A5533) for identification of osteoblastic differentiation.

PC3 cells were purchased from American Type Culture Collection (ATCC), and cultured in Ham's F‐12K (Kaighn's) medium (Gibco, 21 127 022) with 10% FBS (Gibco, 10099–141C), 2 mm L‐glutamine and 1% penicillin and streptomycin (Gibco, 15 140 122) at 37°C with 5% CO_2_.

### Materials

LDN193189 (HY‐12071), U0126 (HY‐12031), LGK974 (HY‐17545), and recombinant BMP2 protein (HY‐P7006) were purchased from MedChemExpress. TEMPO (426 369), α‐tocopherol (α‐T, T1539), rotenone (R8875), diphenyliodonium chloride (DPI, 43 088), potassium iodide (KI, 221 945), quinacrine dihydrochloride (QCDC, 222 992) were purchased from Sigma–Aldrich. Recombinant human noggin protein (6057‐NG) was purchased from R&D Systems. DTT (ST041) was purchased from Beyotime. Tween‐20 (A100777) was purchased from Sangon Biotech. Cells were treated with these reagents according to the protocols provided by the manufacturers. After the laser stimulation experiments, cells were incubated with these reagents for another 30 min before replacing the cell culture medium. While in BMP2 or DEX induction groups, cells were treated with them during the entire experiments.

The Ca^2+^‐free medium followed this formula (140 mm NaCl, 5 mm KCl, 1 mm MgCl_2_, 10 mm glucose, 10 mm HEPES, 200 mm EGTA, pH 7.4). For further intracellular Ca^2+^ depletion, cells were treated with membrane‐permeable Ca^2+^ chelator BAPTA‐AM (10 µm, Invitrogen, B6769) in Ca^2+^‐free medium for 15 min before experiments.

Cells were incubated with 2 µm Fluo‐4/AM (ThermoFisher, F14202), 5 µm Dihydroergotamine (DHE, Beyotime, S0063), and 5 µg ml^−1^ JC‐1 (Invitrogen, T3168) for 30 min at 37°C, then washed and replaced with clean medium to fluorescently indicate intracellular Ca^2+^, ROS, and MMP, respectively. The green and red fluorescence signals were excited at 473 and 543 nm and detected in spectral bands of 500–530 nm and 553–618 nm, respectively.

### Photoactivation Scheme

A Yb‐doped fiber femtosecond laser (1030 nm, ≈220 fs, 1 MHz, MenloSystems, BlueCut) and a Ti: Sapphire femtosecond laser (tunable from 680–1080 nm, 140 ± 20 fs, 80 MHz, Chameleon Ultra II, Coherent) were coupled into an inverted confocal microscope (FV1200, Olympus). The femtosecond laser shared the same light path with the confocal microscopy for simultaneous photostimulation in any predefined region and continuous microscopy of the FOV. The laser beams were controlled by the galvo mirrors (for scanning in the predefined region) and a mechanical shutter that was synchronized. In experiments, the lasers were focused by a 30 ×, 1.05 NA, oil immersion objective. The focused femtosecond laser scanned the defined photostimulation region point by point for photoactivation. Its scanning plane was vertically tuned to the upper half (close to the top) of cells to avoid the laser passing through the cytoplasm.

The photoactivation was defined as a single microscopy frame, controlled by both galvo mirrors and the shutter, which could be inserted in a pre‐defined time‐lapse confocal microscopy sequence for real‐time microscopy and photoactivation. The photostimulation duration was thus the time of the frame.

Cells adherent on glass‐bottom (0.17 mm thick) dishes were observed by confocal microscopy and subjected to femtosecond‐laser scanning point by point for a single time in a predefined costume region in FOV. In the FRET experiments, cells were stimulated by the laser scanning in a predefined area (26 × 26 µm^2^) in the FOV (420 × 420 µm^2^) frame‐by‐frame (40 × 40 pixels, 2 µs/pixel, 0.06 s/frame) for 0.5 s at 10 mW using the femtosecond laser at 1030 nm. In other experiments, cells were stimulated by the laser at 1030 nm scanning in the whole FOV (420 × 420 µm^2^) for a single frame (640 × 640 pixels, 2 µs/pixel, 1.5 s) at 8 mW. For the BMP activation efficiency, the tunable femtosecond laser was used to activate cells in a predefined region (26 × 26 µm^2^) frame‐by‐frame (40 × 40 pixels, 2 µs/pixel, 0.06 s/frame) for 5 s. The power of 700–1000 nm laser at the specimen was kept at 30 mW.

### siRNA and Plasmids Transfection

Synthetic small interference RNA (siRNA) sequences targeting BMP receptors type Ia was synthesized and purchased from Genomeditech, Shanghai. siRNA with a nontargeting sequence (siNC) was used as a negative control.

Plasmids of YFP‐BMPR1a, CFP‐BMPR2, and BMPR1a‐YFP were cloned and purchased from Tsingke Biotech, Beijing. BMPR2‐CFP and YFP‐BMPR1a‐C180S were cloned and purchased from GeneChem, Shanghai. The DNA fragments encoding fluorescent proteins (YFP and CFP) were inserted in‐frame to the N‐terminus or C‐terminus of DNA sequences encoding for the BMP receptors type Ia, II (downstream of the signal peptide encoding sequence when inserted to N‐terminus), respectively. YFP‐BMPR1a‐C180S was a mutation of YFP‐BMPR1a, with BMPR1a protein cysteine 180 mutated to serine (C180S). PCMV‐N‐YFP (D2716) was purchased from Beyotime and used as whole‐cell YFP fluorescence control.

Cells were transiently transfected with the siRNAs or plasmids through jetPRIME (Polyplus‐transfection) according to the manufacturer's protocol. Cells were prepared 24 h after the transfection for laser stimulation or silencing validation.

### FRET Assay

FRET experiments were performed on cells co‐transfected simultaneously with (YFP‐BMPR1a and CFP‐BMPR2 plasmids) or (BMPR1a‐YFP and BMPR2‐CFP plasmids). The excitation wavelength of CFP (donor) was 405 nm. The excitation and bleaching wavelength of YFP (acceptor) was 473 nm.

We measured the CFP fluorescence intensity of the target cell at first, then bleached the acceptor YFP in this cell by continuous scanning of the 473 nm laser, and recorded CFP fluorescence intensity after photobleaching. To test the extent of photoactivated conformational changes of BMPRs, after recording the CFP fluorescence baseline, cells were photostimulated and the CFP fluorescence was then measured. The YFP was then bleached, and the CFP fluorescence was measured again. The CFP (donor) fluorescence fluctuations indicated the original transferred energy to YFP (acceptor), which thus reflected the efficiency of FRET.

### Microscopy and Immunofluorescence Labeling

A confocal microscope (FV1200, Olympus) was used for confocal microscopy. Confocal images were usually acquired with 1024 × 1024 pixels/frame at 2 µs/pixel and further processed by ImageJ.

Primary antibodies, anti‐phospho‐Smad1/5/8 rabbit antibody (Cell Signaling Technology, 13 820, 1:800), anti‐Runx2 rabbit antibody (Cell Signaling Technology, 12 556, 1:5000), anti‐BMPR1a rabbit antibody (ThermoFisher, 38–6000, 2 µg mL^−1^), anti‐phospho‐Smad2 rabbit antibody (Cell Signaling Technology, 18 338, 1:800), anti‐phospho‐p38 rabbit antibody (Cell Signaling Technology, 4511, 1:1600), anti‐phospho‐eIF4E rabbit antibody (Abcam, ab76256, 1:500), anti‐beta Catenin rabbit antibody (Abcam, ab32572, 1:500), were used according to the protocols from manufacturers.

Cells were first cultured in 35 mm glass‐bottom (0.17 mm thick) dishes. For immunofluorescence microscopy, cells were fixed with 5% paraformaldehyde (Beyotime) and permeabilized with 0.1% Triton X‐100 (Beyotime). After being blocked with 1% bovine serum albumin (Sigma–Aldrich), cells were incubated with primary antibodies at 4°C overnight. The sample was washed with PBS for 5 min, and then incubated with secondary antibodies Goat anti‐Rabbit IgG H&L‐Green (Alexa Fluor 488) (Abcam, ab150077, 1:1000) for 1.5 h at room temperature.

### Western Blotting

Cells were dissected and homogenized in lysis buffer (RIPA: protease inhibitor cocktail = 100:1). Whole cell lysates were analyzed in a western blot experiment according to standard protocols. Antibodies used in immunoblotting included anti‐BMPR1a rabbit polyclonal antibody (ThermoFisher, 38–6000, 2 µg mL^−1^), anti‐GAPDH mouse monoclonal antibody (Proteintech, 60004‐1‐Ig, 1:5000), Peroxidase AffiniPure goat anti‐rabbit IgG (H + L) (Jackson ImmunoResearch, 111‐035‐003, 1:10 000), and Peroxidase AffiniPure goat anti‐mouse IgG (H + L) (Jackson ImmunoResearch, 115‐035‐003, 1:10 000). Proteins were visualized using enhanced chemiluminescence. The images were acquired by Tanon 4200SF (Tanon).

### Covalent Binding Site Screening

For the covalent binding Cys site screening analysis of flavin molecules and BMPR1a, flavin molecule was downloaded from the PUBCHEM based on CAS number and BMPR1a from the UniProt database. The structure of BMPR1a was evaluated for reasonableness using the protein structure evaluation function of MOE2015. The results showed that three amino acid residues (AN16, HIS31, SER28) were located in the impermissible region of the protein structure with a percentage of 0.6% (3/532), indicating that the protein structure could be used for molecular docking to screen for Cys amino acid residues that could form a thioether bond with the flavin molecule. Covalent docking experiments were performed using MOE2015 software. The docked Cys site was used as the reaction site, the reaction type was Michael acceptor, and other settings were used as parameters for molecular docking. The covalent docking results of all sites were counted and the binding possibility was judged based on the binding free energy. The results of each Cys in BMPR1a are shown in the following table.

**Table:** 

Cys site	Binding free energy [kcal mol^−1^]
180	−4.4087
503	−4.1246
173	−3.7511
323	−3.6052
14	−3.5729
376	−3.387
130	−3.3829
125	−3.3618
67	−3.3459
377	−3.3126
163	−3.3114
340	−3.3102
175	−3.3039
61	−3.1768
475	−2.9428
110	−0.451
63	/
76	/
82	/
100	/
124	/
343	/
449	/
492	/

### Statistics

All experiments were performed for at least three independent times. The sample size n was provided in each figure legend accordingly. The images were processed by ImageJ. Statistical analysis and graphs were conducted with GraphPad Prism 9. Statistical significance was calculated using two‐tailed unpaired *t*‐test or one‐way ANOVA analysis corrected by Dunnet's post‐hoc multiple comparison tests.

The data were presented as the means ± standard error of the mean (SEM). The confidence interval was set as 95%.

## Conflict of Interest

The authors declare no conflict of interest.

## Author contributions

H.H. conceived the study and supervised the project. M.X. performed the experiments and prepared the figures. H.W. helped with the optical setup. X.T. helped the plasmids and transfection. H.H. drafted the manuscript. All authors analyzed the data, discussed the results, and revised the manuscript.

## Supporting information

Supporting Information

## Data Availability

The data that support the findings of this study are available from the corresponding author upon reasonable request.
