# Supplementary material for: Opsin‐Free Activation of Bmp Receptors by a Femtosecond Laser
Source: Adv Sci (Weinh). 2024 Jan 18;11(10):2308072. doi: 10.1002/advs.202308072 (PMC10933676; doi:10.1002/advs.202308072)
Supplement: Supplementary file 1 — Supporting Information [file ADVS-11-2308072-s001.pdf]

ADVANCED SCIENCE
Open Access

Supporting Information

for *Adv. Sci.*, DOI 10.1002/advs.202308072

Opsin-Free Activation of Bmp Receptors by a Femtosecond Laser

*Manjun Xu, Haipeng Wang, Xiaoying Tian, Bingyi Li, Shaoyang Wang, Xiaohui Zhao and Hao He**

Supplementary Figures

Manjun Xu[1], Haipeng Wang[1], Xiaoying Tian[1], Shaoyang Wang[2], Bingyi Li[1], Xiaohui Zhao[1], Hao He[1,*]

[1]School of Biomedical Engineering, Shanghai Jiao Tong University, Shanghai, China

[2]School of Biomedical Engineering, Hainan University, Haikou, China

*Corresponding author. E-mail: haohe@sjtu.edu.cn

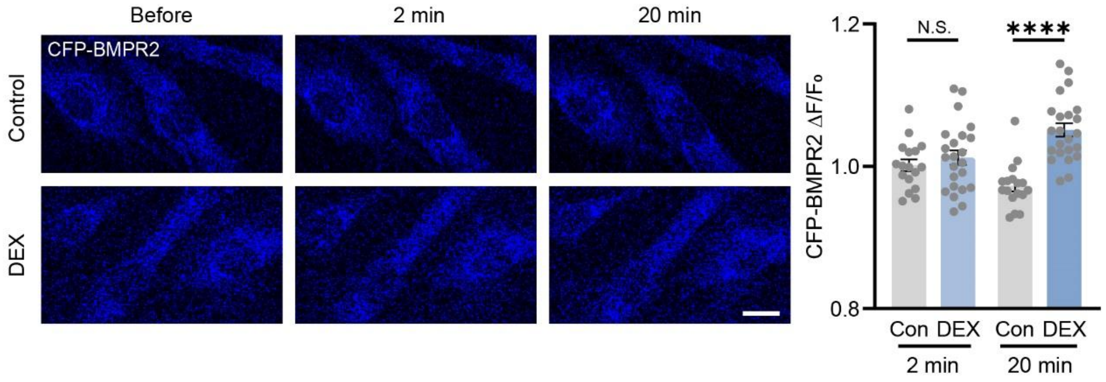

Fig. S1. CFP-BMPR2 and YFP-BMPR1a FRET test indicated by CFP fluorescence after DEX treated (n=23 cells in 3 independent trials). Control: ADSCs without any treatment (n=17 cells in 3 independent trials). Scale bar: 20 μm. Right panel: quantified CFP fluorescence after DEX treated. ****$P < 0.0001$ by two-tailed unpaired t test. N.S., no significant difference.

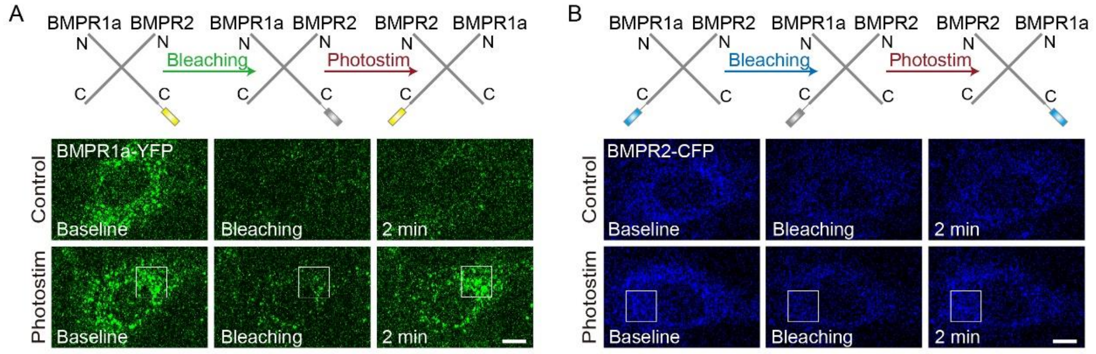

Fig. S2. Pre-bleached BMPR1a-YFP (A) and BMPR2-CFP (B) recovered after photostimulation. Boxes, photostimulation region. Scale bar: 10 μm.

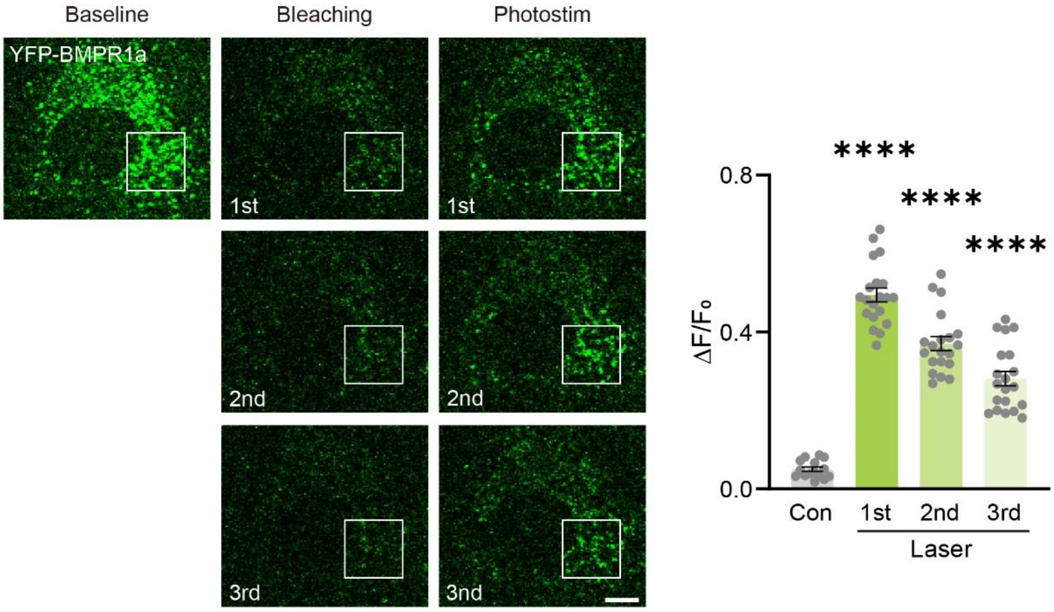

Fig. S3. BMPR1a-YFP recovered after photobleaching and photostimulation (n=20 cells in 3 independent trials) for multi-times. Boxes, photostimulation region. Scale bar: 10 μm. Right panel: quantified recovered ratio of YFP fluorescence. ****P < 0.0001 by one-way ANOVA analysis corrected by Dunnet's post-hoc.
